# Unraveling the relationship between stress exposure and childhood anxiety: Considering accumulation, impact, and type in the first five years of life

**DOI:** 10.1371/journal.pone.0315019

**Published:** 2024-12-26

**Authors:** Viviane Valdes, Linda W. Craighead, Charles A. Nelson, Michelle Bosquet Enlow

**Affiliations:** 1 Boston Children’s Hospital (Division of Developmental Medicine), Brookline, Massachusetts, United States of America; 2 Harvard Medical School (Department of Pediatrics), Boston, Massachusetts, United States of America; 3 Emory University (Department of Psychology), Atlanta, Georgia, United States of America; 4 Harvard Graduate School of Education, Cambridge, Massachusetts, United States of America; 5 Boston Children’s Hospital (Department of Psychiatry and Behavioral Sciences), Boston, Massachusetts, United States of America; 6 Harvard Medical School (Department of Psychiatry), Boston, Massachusetts, United States of America; Isfahan University of Medical Sciences, ISLAMIC REPUBLIC OF IRAN

## Abstract

Exposure to stressful events is linked to anxiety symptoms in children, although research examining this association in the first five years of life is limited. We sought to examine the role of various aspects of family stressful experiences such as the total accumulation, impact, and type (measured longitudinally in the first five years of life) on child anxiety symptoms at age 5 years. A community sample of children and their parents (N = 399) enrolled in a longitudinal study of emotion processing were assessed when the children were infants and at ages 2 years, 3 years, and 5 years. Parents completed the Revised Life Events Questionnaire (all visits) to assess family exposures to stressful life events and the Child Behavior Checklist (5 years) to assess child emotional and behavioral symptoms. Analyses showed that total stressful events accumulated by 4 years were significantly associated with child anxiety symptoms at 5 years (*r* = 0.118, *p* = 0.045). Total stressful events accumulated at earlier time points (by 1 year, 2 years, and 3 years of age) were not significantly associated with child anxiety symptoms at 5 years. Events reported as being impactful by families appeared to be more sensitive than total events, with accumulated impactful events as early as 1 year being significantly associated with child anxiety symptoms at 5 years (*r* = 0.112, *p* = 0.042). When considering types of stressors, cumulative exposure from the prenatal period to 5 years to financial stressors (*β* = 0.12, *p* = .035) was most saliently and significantly associated with child anxiety symptoms at 5 years (after adjusting for other categories of stress such as health, interpersonal, and logistical stressors). Together, these findings suggest that stressful life events accumulated in early life, particularly those rated as impactful for the family and those related to finances, are associated with child anxiety symptoms at 5 years.

## Introduction

The Centers for Disease Control and Prevention (CDC) estimates that 7.1% of 3- to 17-year-old children in the U.S. have a diagnosed anxiety disorder [1]. Anxiety disorders in childhood predict impairment in functioning later in life in a variety of domains (*e*.*g*., physical, financial, interpersonal) [2]. In terms of burden, the World Health Organization (WHO) estimates that mental health disorders account for over 175 million years of productive life lost to disability (YLDs) in 2010, making it the leading cause of YLDs [3]. Anxiety disorders account for almost 15% of those years and are the sixth leading cause of YLD [3,4]. Given the significant prevalence and burden of anxiety disorders across the lifespan, identifying risk and protective factors has been an area of focus for research to inform intervention efforts [5–8].

Exposure to stressful life events has been studied as a potentially important risk factor for anxiety and mood disorders. Stressful life events related to loss are believed to be more specific to the development of depression in school aged children, whereas events involving threat may be more closely linked to anxiety disorders [9,10]. Existing research also suggests that a greater number, severity, and chronicity of stressful life events is associated with the onset of anxiety in school aged children [11,12]. Exposure to stressful life events also been found to be associated with physical and social anxiety symptoms (stress causation hypothesis); in contrast, there is less evidence that anxiety leads to exposure to later stressors in childhood and adolescence (stress generation hypothesis) [13,14]. McLaughlin and Hatzenbuehler (15) found, using a longitudinal design in a diverse sample, that stressful life events were associated with increased anxiety symptoms in adolescents. They further found that certain types of stressful events, such as those related to health and family discord, better accounted for anxiety symptoms than other types of events in adolescence [15].

The extant literature provides compelling evidence for stressful life events within the family environment as a broad correlate of childhood anxiety symptoms and identifies several other parent and child characteristics that correlate with child anxiety symptoms/disorders. Most of these studies are in school-aged children, adolescents, and young adults. Moreover, these studies have typically examined bivariate associations (*e*.*g*., between interpersonal stressors and anxiety symptoms) in cross-sectional studies. There is a gap in our understanding of how different aspects or levels of stressful experiences (e.g., timing of total accumulation, events that are rated as being impactful, category or type of stressful experience) might differentially affect risk for childhood anxiety. This is especially true in early life, when stress regulatory systems are rapidly developing and susceptible to the programming effects of stress exposures [12,16–19]. The current study aims to address some of these gaps in the literature and contribute to the growing body of work elucidating risk factors for childhood anxiety.

Specifically, we sought to determine: 1) At which incremental point of accumulation does exposure to a greater total number of familial stressful life events (i.e., by 1 year, 2 years, 3 years, 4 years, and 5 years of life) become associated with anxiety symptoms in children at age 5 years; 2) whether impactful stressful events may be a more sensitive metric and be associated with child anxiety symptoms at earlier ages; 3) which type of stressful event exposure across four categories of stressful experiences (health, interpersonal relationships, finances, logistics) is most saliently associated with anxiety symptoms at 5 years.

## Methods

### Participants

Participants were recruited from a registry of local births comprising families who had indicated willingness to participate in developmental research. Families in the current analyses are part of a longitudinal study originally designed to examine the early development of emotion processing. Exclusion criteria in the parent study included known prenatal or perinatal complications, maternal use of medications during pregnancy that may have significant impact on fetal brain development (i.e., anticonvulsants, antipsychotics, opioids), pre- or post-term birth (±3 weeks from due date), developmental delay, uncorrected vision difficulties, and neurological disorder or trauma. After initial enrollment at infancy, families were excluded from the study and no longer followed for additional assessments if their child was diagnosed with a genetic or other condition known to influence neurodevelopment (*n* = 29).

By design, families were enrolled in the parent study when the children were 5, 7, or 12 months old (infancy), with a smaller subsample to be followed when the children were 2 years, 3 years, and 5 years of age. Approximately 778 infant-mother dyads were enrolled in the baseline visit but only participants with child anxiety data at the 5-year visit were included in the current analyses (*N* = 399). Independent-sample proportions tests were run to determine whether proportions across several demographic variables at infancy (child’s sex, child’s ethnicity, child’s race, parent education, parent marital status, and annual household income) differed between those retained at 5 years and those lost to follow-up. No significant differences (p < .05) were observed. Study procedures were approved by the Institutional Review Board at Boston Children’s Hospital, and parents or legal guardians provided written informed consent prior to the initiation of study activities. Written assent was also obtained from the child if possible, or the study investigators indicated in consent forms that the child was too young to provide written assent. Participants were recruited for the initial visit in infancy between April 23, 2013 and April 24, 2017.

### Measures

Questionnaires, described below, were administered via REDCap, an online platform, to the child’s parent, primarily the child’s mother (97%).

#### Demographics

Demographic information was collected via parent-report at infancy to characterize the sample. The following demographic information was collected: child age at each visit, sex assigned at birth (hereafter “sex”; male/female), ethnicity (non-Hispanic/Latinx, Mexican, Puerto Rican, Cuban, Other Hispanic/Latinx, or Mixed Hispanic/Latinx), race (White, Black or African American, American Indian or Alaska Native, Asian Indian, East Asian, Pacific Islander, Mixed Race), and parent education (8^th^ grade or less, some high school, high school/GED, associate’s degree, bachelor’s degree, master’s degree, doctoral degree).

#### Family exposure to stressful life events

Exposure to stressful life events was measured at each assessment time point using a 30-item version of the Recent Life Events Questionnaire (RLEQ; [20]. The RLEQ was developed to identify common life events that a high proportion of respondents report as having marked or moderate long-term threat (as opposed to mild or no long-term threat). The RLEQ includes items pertaining to serious injury or illness, death of relative or friends, separation (relationship or marriage), serious problems with close friends or relatives, serious abuse/attack/threats, unemployment or job loss, financial crisis, problems with police or court appearance, burglary/mugging, miscarriage/stillbirth, moving homes (through choice or not), and housing difficulties. For example, one of the items asks, “Have you had any major financial difficulties (e.g. debts, difficulty paying bills)?” and another asks, “Have you witnessed violence or threats of violence in your neighborhood or community?”.

The parent was asked to rate whether they had experienced the event (yes/no) in the time period of interest (e.g., from pregnancy to the time of the infant assessment) and how much each endorsed event had affected their life since the event occurred (on a Likert scale “not at all” = 0, “a little bit” = 1, “medium” = 2, “a lot” = 3). Two scores were calculated: (a) a sum of the endorsed events, i.e., the number of stressful experiences reported over the time period assessed; and (b) an impact score, i.e., the sum of scores asking how much each endorsed event had affected their life since the event occurred. In addition, summary scores for four specific categories of stressful events (health, interpersonal relationships, finances, logistics) were tabulated. Some examples of events by category include illnesses or injuries (health), problems with close friends (interpersonal), job loss or unemployment (financial), and moving homes or housing difficulties (logistics).

The questions for the RLEQ were modified such that the parent was asked to report on events since the prior questionnaire administration (or from the time the respondent learned of the pregnancy until the time of assessment in the case of the infancy assessment); this allowed for calculation of cumulative scores for exposure to stressful events over the entirety of the child’s life. Scores from pregnancy to 1 year, pregnancy to 2 years, pregnancy to 3 years, pregnancy to 4 years, and pregnancy to 5 years of life were used (measured at infancy visit, 2-year visit, 3-year visit, and 5-year visit, respectively).

#### Child anxiety

Child anxiety was measured at age 5 years using the parent-report Child Behavior Checklist (CBCL) for ages 1.5 to 5 years [21,22]. The CBCL 1½-5 assesses behavioral and emotional problems in young children. The parent-report version obtains parent ratings for 99 problem items that can be scored according to syndrome or DSM-oriented scales [23]. Parents were asked to rate how true each item was for their child in the prior 6 months on a 3-point scale (“not true” = 0, “somewhat or sometimes true” = 1, “very true or often true” = 2). The current analyses used the DSM-oriented “anxiety problems” T-score scale as the assessment of child anxiety symptoms [23]. T-scores on the CBCL have a mean of 50 and a standard deviation of 10. Scores in the 60–63 range are considered borderline for psychopathology, and scores greater than 63 are considered clinically elevated. The internal consistency estimate for the current sample on the anxiety problems scale was α = .69.

#### Data analysis plan

Data analyses were conducted using IBM SPSS Statistics for Macintosh Version 28 (IBM Corp, Armonk, NY, USA). Descriptive statistics for demographic variables were run in addition to the main analyses. Data were evaluated for normality and multicollinearity using univariate procedures and tolerance statistics. Bivariate analyses were conducted to identify potential covariates that should be included in models. Covariates that were significantly associated with the main exposure variables, the outcome variable, and did not negatively impact model fit statistics were included in final models. Main analyses tested the study aims as follows:

*Cumulative Stressful Events and Child Anxiety*. Scores for the total number of cumulative stressful events from pregnancy to 1 year, pregnancy to 2 years, pregnancy to 3 years, pregnancy to 4 years, and pregnancy to 5 years of life were used for the analysis (measured at infancy visit, 2-year visit, 3-year visit, and 5-year visit, respectively). Pearson’s correlations were conducted to determine the association between the total number of stressful events reported by each time point and anxiety symptoms at age 5 years. Benjamini-Hochberg (BH) corrections for multiple comparisons were used.

*Impactful Stressful Events and Child Anxiety*. Scores for the impact of stressful life events accumulated by 1 year, 2 years, 3 years, 4 years, and 5 years were used to address this aim. Pearson’s correlations were conducted to determine the association between the impact of endorsed events accumulated by each time point and anxiety symptoms at age 5 years. Benjamini-Hochberg (BH) corrections for multiple comparisons were used.

*Type of Stressful Event and Child Anxiety*. Scores for the impact of specific types of familial stressful events (health, interpersonal relationships, finances, logistics) experienced between the prenatal period and age 5 years were used for this analysis. A linear regression model was used to determine the relative contribution of each category of stressful life events on child anxiety symptoms at age 5 years. Standard beta coefficients were used to determine the relative contribution of each type of stressor to the outcome.

## Results

### Preliminary analyses

Descriptive statistics demonstrating the sample’s demographic characteristics are presented in Table 1. Descriptive statistics for the main study variables are reported in Table 2. At the five year visit, 14.3% (*n* = 57) of the sample was in the borderline or clinical range for anxiety.

**Table 1:** Sample demographics *(N* = 399).

Variables	*n*	*%*	*M (SD)*
Age at infant visit (in months) Age at 2-year visit (in months) Age at 3-year visit (in months) Age at 5-year visit (in months) Child sex Male Female Child race White Black/African American Asian American or Pacific Islander Mixed race Not reported Child ethnicity Non-Latino/a, Spanish, or Hispanic Mexican Puerto Rican Cuban Other Latino/a, Spanish, or Hispanic Mixed Latino/a, Spanish, or Hispanic Not reported Maternal educational attainment Less than high school High school/GED Associate degree Bachelor’s degree Master’s degree M.D., Ph.D., J.D., or equivalent Not reported Paternal educational attainment Less than high school High school/GED Associate degree Bachelor’s degree Master’s degree M.D., Ph.D., J.D., or equivalent Not reported Annual family household income Less than $35,000 $35,000-$49,000 $50,000-$74,999 $75,000-$99,000 $100,000 or more Not reported	210 189 315 6 10 62 6 360 6 4 2 21 2 4 0 13 7 116 183 79 1 0 39 18 115 125 99 3 10 11 38 62 241 37	52.6 47.4 78.9 1.5 2.5 15.5 1.5 90.2 1.5 1.0 0.5 5.3 0.5 1.0 0 3.3 1.8 29.1 45.9 19.8 0.3 0 9.8 4.5 28.8 31.3 24.8 0.8 2.6 2.8 9.5 15.5 60.4 9.3	7.74 (3.03) 24.17 (1.36) 37.22 (4.66) 62.02 (2.13)

**Table 2:** Descriptive statistics for stressful life events and child anxiety symptoms.

Variable	5-year visit *M (SD)*
Stressful life events (RLEQ) Cumulative events (infancy to 5 years) Cumulative impact (infancy to 5 years) Child Anxiety T-Score (CBCL 1½-5) T-score	11.70 (7.08) 16.56 (14.78) 53.24 (5.95)

Bivariate analyses of the main study variables with all demographic variables (child sex, child race, child ethnicity, parent education, family income) were conducted to identify potential covariates. None of the demographic variables tested were significantly associated with any of the variables of interest and thus were not included in subsequent models. Stressful event scores across time were significantly correlated with each other, with correlation coefficients ranging from .784 to .881 and p-values all < .001.

#### Cumulative stressful events and child anxiety

Pearson’s correlations were conducted to identify at which time point (by 1 year, 2 years, 3 years, 4 years, or 5 years) accumulation of stress becomes associated with subsequent anxiety symptoms (Table 3). Cumulative events by the 4 years (*r* = 0.118, *p* = 0.045) and by 5 years (*r* = 0.148, *p* = 0.015) were significantly associated with child anxiety symptoms at 5 years of age after adjusting for multiple comparisons. Total accumulated stressful life events by 1 year, 2 year, and 3 years of age were not significantly associated with child anxiety symptoms at 5 years.

**Table 3:** Associations between cumulative total stressful life events and child anxiety symptoms.

Variable	*r*	*n*	*p* *(uncorrected)*	*p* *(corrected)*
Total events (prenatal to 1 year)	.078	396	.123	0.154
Total events (prenatal to 2 years)	.061	396	.229	0.229
Total events (prenatal to 3 years)	.089	395	.077	0.128
Total events (prenatal to 4 years)	.118	399	.018	0.045
Total events (prenatal to 5 years)	.148	399	.003	0.015

*Note*. P-values corrected using the Benjamini-Hochberg (BH) procedure.

#### Impactful stressful events and child anxiety

Pearson’s correlations were also conducted to test the next aim seeking to determine whether exposure to impactful stressful events may be a more sensitive metric of stress, with associations observed between accumulated scores and anxiety symptoms at 5 years at earlier time points (Table 4). For events that were reported as having an impact, associations were significant after correcting for multiple comparisons as early as the first year of life (*r* = 0.112, *p* = 0.042). Almost every time point of accumulation (impactful events by 1 year, 3 years, 4 years, and 5 years) was significantly associated with child anxiety symptoms at 5 years; impactful events that were accumulated by year 2 had a similar effect size (*r* = 0.095) but only approached significance (*p* = 0.060).

**Table 4:** Associations between cumulative impactful stressful life events and child anxiety symptoms.

Variable	*r*	*n*	*p* *(uncorrected)*	*p* *(corrected)*
Impactful Events (prenatal to 1 year)	.112	396	.025	0.042
Impactful Events (prenatal to 2 years)	.095	396	.060	0.060
Impactful Events (prenatal to 3 years)	.105	392	.037	0.046
Impactful Events (prenatal to 4 years)	.133	398	.008	0.020
Impactful Events (prenatal to 5 years)	.153	399	.002	0.010

*Note*. P-values corrected using the Benjamini-Hochberg (BH) procedure.

#### Stressful event type and child anxiety

A linear regression model was used to determine the relative contribution of stressful life event categories (finances, health, interpersonal relationships, logistics) on child anxiety symptoms at age 5 years. Stressful events by category included cumulative reports from infancy to 5 years of age. Collinearity statistics (tolerance and variance inflation factor) were run for this model, given that each of the categories were collected using the same measure and thus may be correlated, affecting the regression model’s findings. Tolerance statistics ranged from .788 to .952, and VIF statistics ranged from 1.051 to 1.269. Typically, values of VIF (= 1/tolerance) exceeding 10 are believed to indicate multicollinearity, although some maintain that values near 2.5 may be a cause for concern and indicate moderate levels of collinearity [24].

Given that all VIFs were well below 2.5 and correlations across categories were weak, we concluded that variables in the current linear regression model likely did not have significant multicollinearity. A linear regression model was run to determine which categories best contributed to child anxiety. Full results are presented in Table 5. Cumulative financial stressors (*β* = .122, *p* = .035) was the only category that was significantly associated with child anxiety at age 5 years. Other categories (health, interpersonal relationships, logistics) were not associated with the outcome in regression models.

**Table 5:** Type of stressful event (cumulative by 5 years) and child anxiety symptoms at 5 years.

Category	B (SE)	β (standardized)	*p*	Tolerance, VIF
Finances	.259 (.122)	.122	.035	.788, 1.269
Health	.062 (.088)	.036	.484	.952, 1.051
Interpersonal relationships	.079 (.146)	.029	.590	.845, 1.184
Logistics	.076 (.126)	.033	.547	.833, 1.201
R^2^	.025			
Adjusted R^2^	.015			
F	2.552			

*Note*. Category scores from the Recent Life Events Questionnaire (RLEQ).

## Discussion

The goal of the present study was to examine whether stressful family life events experienced during the child’s first five years of life increased risk for anxiety symptoms assessed at 5 years of age. Results indicated that the total number of stressful life events accumulated by 4 and 5 years of life were associated with child anxiety symptoms at 5 years of age. When analyses considered the parent-reported impact of the stressful life events experienced, events accumulated as early as infancy were associated with child anxiety symptoms at 5 years. In analyses that considered separate categories of events (financial, health, interpersonal relationships, logistics), financial stressors were most saliently associated with child anxiety symptoms.

In prior work, a greater number, severity, and chronicity of stressful life events has been associated with the onset of anxiety in children ages 6–12 years [11]. The current findings are consistent with such research, suggesting that stressful life events accumulated over the first five years of life are associated with anxiety symptoms in children by 5 years. The current findings add to the extant literature by demonstrating that stressors accumulated up to time points closer in time to the anxiety assessment (here, prenatally to 4 and 5 years of life) may be more strongly linked to anxiety symptoms at 5 years than those accumulated earlier (prenatally to the 1, 2, and 3 years respectively). This pattern of findings may be due to the greater accumulation of stressful experiences by the time children reach later developmental timepoints. It is also possible that this stronger association is due to proximity in assessment between stressful experiences accrued by 4–5 years of age and child anxiety at 5 years of age.

Notably, when stressful life event impact scores were analyzed, cumulative stressors as early as 1 year of life were associated with child anxiety symptoms at 5 years, indicating the long-term potential impact on child mental health of family negative life events even in infancy. These findings suggest that exposures to events generally considered to be potential stressors may be less associated with child psychological functioning than assessing for events that the families reported as having meaning or impact for them, possibly due to ongoing psychological stress experienced as a result of the exposure. Thus, assessing for exposure to events believed to be generally stressful to families may be less useful than using a score that includes the family’s assessment of whether those events impacted their functioning.

Prior studies have explored the link between types of stressful life events experienced by families and anxiety symptoms in children and adolescents. Some studies suggest that life events related to loss may be more highly associated with depressive symptoms in children (6–12 years of age), whereas threat events may be more closely linked to anxiety [9,10]. Stressful events related to health and family discord may account for greater increases in anxiety sensitivity in adolescents [15]. Additionally, in adolescents, exposure to stressors related to peer relationships is associated with increased anxiety [25]. In the current study, stressful events related to finances over the first 5 years of life were more robustly related to child anxiety symptoms than events related to health, logistics, or interpersonal relationships.

It may be the case that the type of stressor that is most significantly related to anxiety symptoms varies as a consequence of developmental timing. Financial stressors may have a greater impact earlier in life, as they may disrupt the provision of basic needs, caregiving quality, and household climate, the main routes by which very young children are likely to experience stress. In contrast, at later developmental time points (e.g., adolescence), exposure to different types of stressors may be related to anxiety outcomes as evidenced by work by McLaughlin and Hatzenbuehler [15]. Early childhood is characterized by a dependence on the immediate family environment and financial stressors during this period may be more linked to disruptions that produce anxiety [26–28]. On the other hand, adolescents tend to have expanded social environments and more complex awareness of emotional processes, which may make their anxiety symptoms more susceptible to stressors that are interpersonal in nature (both with family and peers).

There are several proposed mechanisms through which financial stressors and being in a lower to middle income group more generally, may influence mental health outcomes in adults. These include increases in worries and uncertainty, exposure to environmental irritants (air pollution, sleep deprivation), worse physical health outcomes, increased risk for exposure to direct threats (*e*.*g*., to intimate partner violence or violent crimes), and greater degrees of shame (*e*.*g*., through social comparison) and isolation [29]. Many of the mechanisms that mediate associations between financial stressors and mental health outcomes experienced by adults are co-occurring for children. Additionally, these mechanisms may affect the quality of caregiving, safety, and stability of early environments. An existing body of research is beginning to document the impact of poverty and financial stressors on brain and cognitive development in early childhood [30,31]. Additional research is needed to further elucidate mechanisms for child anxiety symptoms, as well as other aspects of mental health, in the first five years of life when the brain may be particularly plastic and susceptible to environmental stressors [32–35].

### Strengths and limitations

The current study has notable strengths, including a longitudinal design that allowed for the investigation of timing effects, a large sample size, data on stressors with multiple operationalizations to better understand effects (*e*.*g*., total stressors, impact of stressors, types of stressors, timing of stressors). However, the current findings should be considered within the context of the study’s limitations. Generalizability may be limited due to the sample’s characteristics, as it was predominantly of middle to upper income, well-educated families living in an urban area of the United States. For instance, in the current sample family household income was not associated with anxiety outcomes in preliminary analyses, possibly due to the restricted range in income levels within the sample. Existing research suggests that lower levels of education, subjective social status, parent employment, neighborhood characteristics, and socioeconomic status (SES) broadly are associated with anxiety disorders, with those in lower SES groups having an increased risk for anxiety [26–28,36–42]. Additional epidemiological research in more socioeconomically diverse samples is needed to determine whether the current findings generalize to other populations and settings. More research in clinical settings is also necessary to determine whether findings from the current community sample hold or differ in those with diagnosed anxiety disorders.

Finally, given the age of participants in the current study (infancy to 5 years), the measures relied on parent report of the child’s environment and behavior, which might introduce bias (e.g., due to parent psychological state) and inflated associations due to the ratings being made by the same respondent. However, recent work suggests that mothers’ psychopathology produces minimal bias in their ratings of their children’s emotions and behaviors [43,44]. It is also notable that there were observed associations between the general stressor measure and child anxiety when assessed as much as 4 years apart, suggesting that associations between stressor and child anxiety cannot be attributed solely to reporting bias of the parent at the time of the completion of the questionnaires (i.e., current psychological state). Nonetheless, maternal psychopathology may influence both the perceived impact of stressors and the subsequent behaviors/environments that ultimately produce anxiety outcomes in children, highlighting the need for future research to investigate interactions between stress exposures and maternal psychopathology at this age.

## Conclusions

Familial exposure to stressful life events as early as infancy was associated with child anxiety symptoms at 5 years in a community sample of typically developing children. The magnitude of association of stressors with child anxiety depended on the nature and familial impact of the stressor. Exposure to potentially stressful experiences accumulated by the time children reached later developmental timepoints (*e*.*g*., by 4 and 5 years of age) were linked with child anxiety symptoms at 5 years of age. When stressful life event impact scores were considered, stressors as early as infancy were associated with child anxiety symptoms at 5 years. These findings suggest that assessing for exposure to events believed to be generally stressful to families may be less useful than using a score that includes the family’s assessment of whether those events impacted their functioning. In terms of exposure types, stress exposures related to finances over the child’s life were most robustly associated with child anxiety symptoms at 5 years in this community sample.
